# The clinical utility of comprehensive measurement of autoimmune disease-related antibodies in patients with advanced solid tumors receiving immune checkpoint inhibitors: a retrospective study

**DOI:** 10.1016/j.esmoop.2022.100415

**Published:** 2022-03-02

**Authors:** N. Izawa, H. Shiokawa, R. Onuki, K. Hamaji, K. Morikawa, H. Saji, H. Ohashi, S. Kasugai, N. Hayakawa, T. Ohara, Y. Sunakawa

**Affiliations:** 1 Department of Clinical Oncology, St. Marianna University School of Medicine, Kawasaki, Japan; 2 Department of Pharmacy, St. Marianna University Hospital, Kawasaki, Japan; 3 Department of Internal Medicine, Division of Respiratory Medicine, St. Marianna University School of Medicine, Kawasaki, Japan; 4 Department of Chest Surgery, St. Marianna University School of Medicine, Kawasaki, Japan; 5 Department of Dermatology, St. Marianna University School of Medicine, Kawasaki, Japan; 6 Department of Otorhinolaryngology, St. Marianna University School of Medicine, Kawasaki, Japan; 7 Department of Urology, St. Marianna University School of Medicine, Kawasaki, Japan; 8 Department of Obstetrics and Gynecology, St. Marianna University School of Medicine, Kawasaki, Japan

**Keywords:** immune checkpoint inhibitors, immune-related adverse events, autoimmune disease-related antibodies

## Abstract

**Background:**

The comprehensive measurement of autoimmune disease-related antibodies (Abs) before immune checkpoint inhibitor (ICI) treatment may be useful for predicting the development of immune-related adverse events (irAEs); however, the clinical utility is not well known.

**Materials and methods:**

We retrospectively analyzed patients with advanced solid tumors treated with ICI monotherapy or doublet combination therapy between July 2014 and December 2020 at single institute. Anti-nuclear antibody (ANA), anti-thyroglobulin (Tg) Ab, anti-thyroid peroxidase (TPO) Ab, anti-glutamic acid decarboxylase (GAD) Ab, anti-acetylcholine esterase receptor (AchR) Ab, and platelet-associated immunoglobulin G (PA-IgG) Ab were comprehensively measured for the screening before ICI therapy.

**Results:**

Of 275 registered patients (median age, 70 years; male, 64.4%; Eastern Cooperative Oncology Group performance status of 0 or 1, 88.7%; and prior regimen of 0-1/≥2, 88.7%/11.3%), 128 non-small-cell lung cancer, 35 gastric cancer, 33 head and neck cancer, 24 melanoma, 19 renal cell carcinoma, 13 urothelial carcinoma, 12 esophageal cancer, 5 malignant mesothelioma of pleura, 2 endometrial cancer, and 4 other cancer were included. The number of patients with positive ANA, Tg, TPO, PA-IgG, GAD, and AchR Abs was 52 (24.9%), 38 (14.5%), 11 (10.1%), 6 (3.5%), 5 (2.0%), and 1 (0.5%), respectively. There was no association between the development of any irAEs and Abs positivity, while thyroid dysfunction developed more frequently among patients with than without Tg Ab or TPO Ab (39.5% versus 12.5%, *P* < 0.01; 45.5% versus 14.3%, *P* = 0.02).

**Conclusions:**

The clinical utility of comprehensive measurement of autoimmune disease-related Abs before introduction of ICI therapy was limited for predicting irAE. However, Tg and TPO Abs were risk factors as regards the development of ICI-induced thyroid dysfunction.

## Highlights

• We analyzed 275 patients with advanced solid tumors treated with the ICI therapy.
• The clinical utility of comprehensive measurement of autoimmune disease-related Abs was evaluated in ICI therapy.
• There was no association between the development of any irAEs and the positivity of Abs.
• Thyroid dysfunction developed more frequently among patients with than without Abs related to the thyroid.

## Introduction

Immune checkpoint inhibitors (ICIs) have rapidly changed the therapeutic landscape, and have been considered as key drugs for patients with cancer. Although serious adverse events from ICI monotherapy are lower compared with cytotoxic chemotherapy, ICI induced a disorder that resembles autoimmune disease, such as ulcerous colitis and fulminant insulin-dependent (type 1) diabetes. The immune-related adverse events (irAEs) led to the permanent discontinuation of ICI. Therefore, it would be necessary to identify the predictors for irAEs.

Menzies et al. demonstrated that the ICI therapy induced frequent immune-mediated toxicities in melanoma patients with preexisting autoimmune diseases before ICI therapy.^1^ Toi et al. also showed that the presence of the examined preexisting antibodies (Abs), such as rheumatoid factor (RF), anti-nuclear antibody (ANA), anti-thyroglobulin (Tg) Ab, and anti-thyroid peroxidase (TPO) Ab before ICI therapy was associated with the development of irAEs in patients with non-small-cell lung cancer (NSCLC).^2^ Particularly, the abnormality of Tg Ab and TPO Ab before ICI therapy may be at high risk of thyroid dysfunction.^3^ In patients diagnosed with type 1 diabetes during ICI therapy, serum anti-glutamic acid decarboxylase (GAD) levels were elevated.^4^ Also, the positivity of anti-acetylcholine esterase receptor (AchR) Ab or platelet-associated immunoglobulin G (PA-IgG) was associated with the onset of myasthenia gravis or thrombocytopenic purpura.^5^^,^^6^ However, there have been only a few data between the presence of those Abs before ICI therapies and the development of irAEs.

The comprehensive measurement of autoimmune disease-related Abs before the ICI treatment would be clinically useful for predicting the development of irAEs; therefore, we routinely carry out comprehensive measurements of immune-related Abs before and during the treatment for cancer patients who receive ICIs. However, it is unclear whether those Abs before ICI therapies will affect the clinical outcomes in patients treated with ICI therapy. This study investigated whether preexisting Abs before introduction of the ICI therapies are predictive factors as regards the development of irAEs during ICI therapy in cancer patients.

## Materials and methods

### Study design and patients

This study included cancer patients with advanced solid tumors treated with ICI, such as anti-programmed cell death protein 1/programmed death-ligand 1 Abs (nivolumab, pembrolizumab, or atezolizumab), anti-cytotoxic T-lymphocyte-associated protein-4 Abs (ipilimumab), or ICI doublet combination therapy (nivolumab and ipilimumab), for the first time between July 2014 and December 2020 at St. Marianna University Hospital. Patients treated with ICI via clinical trials were excluded. This study was approved by the Institutional Review Board of St. Marianna University School of Medicine.

### Assessments

We retrospectively reviewed the medical records of the patients and evaluated clinical information, such as age, sex, Eastern Cooperative Oncology Group (ECOG) performance status (PS), cancer type, history of autoimmune disease, type of ICIs, treatment line, irAEs during ICI therapy, discontinuation of ICI according to irAEs and Abs before and during treatment, ANA, Tg Ab, TPO Ab, GAD Ab, AchR Ab, and PA-IgG. Normal ranges of the Abs in our laboratory were used as a cut-off of 1 : 40 for ANA as previously reported,^7^ less than the criteria value for Tg Ab, <5.61 IU/ml for TPO Ab, <5.0 U/ml for GAD Ab, or negative for AchR Ab and PA-IgG. The irAEs were defined as AEs with a potential immunologic basis and requiring possible intervention with immunosuppressive or endocrine therapy. To reduce bias, only objectively recognizable AEs were considered, such as skin reactions along with endocrine, gastrointestinal tract, hepatic, neurological, and pulmonary events. The clinical severity of the irAEs was graded according to the Common Terminology Criteria for Adverse Events, version 4.0.

### Statistical analysis

This study clarifies the usefulness of the comprehensive measurement of the preexisting Abs before ICI therapy in patients treated with ICIs. We evaluated the association between autoimmune disease-related Abs and irAEs during ICI therapy. The development of irAEs, discontinuation of ICIs, and preexisting Abs positivity were compared using Fisher’s exact test. A *P* value ≤0.05 was considered statistically significant. We conducted all analyses using the JMP version 13.0 (SAS Institute, Cary, NC).

## Results

### Patients’ characteristics

Of 275 patients with advanced solid tumors treated with ICI monotherapy or doublet combination therapy (128 NSCLC, 35 gastric cancer, 33 head and neck cancer, 24 melanoma, 19 renal cell carcinoma, 13 urothelial carcinoma, 12 esophageal cancer, 5 malignant mesothelioma of pleura, 2 endometrial cancer, and 4 other cancer), 193 patients were treated with nivolumab, 67 with pembrolizumab, 10 with atezolizumab, and 5 with nivolumab plus ipilimumab (Table 1). The median age was 70 years (range, 34-91) years, and 244 patients (88.7%) had an ECOG PS of 0 or 1. Fifty-five patients (20.0%) had no prior chemotherapy, whereas 123 patients (44.7%) had received one regimen of chemotherapy, and 97 patients (35.3%) had received two or more regimens.

**Table 1:** Patients’ characteristics

		No. (%) of patients
		*n* = 275
Age, years	Median (range)	70 (34-91)
Sex, *n* (%)	Male/female	177 (64.4)/98 (35.6)
ECOG PS, *n* (%)	0-1	244 (88.7)
	2≤ or ≥2	31 (11.3)
Treatment line, *n* (%)	1	55 (20.0)
	2	123 (44.7)
	3≤ or ≥3	97 (35.3)
Cancer type, *n* (%)		
Non-small-cell lung cancer		128 (46.5)
Gastric cancer		35 (12.7)
Head and neck cancer		33 (12.0)
Melanoma		24 (8.7)
Renal cell carcinoma		19 (6.9)
Urothelial carcinoma		13 (4.8)
Esophageal cancer		12 (4.4)
Malignant mesothelioma of pleura		5 (1.8)
Endometrial cancer		2 (0.7)
Other		4 (1.5)
ICI, *n* (%)		
Nivolumab		193 (70.2)
Pembrolizumab		67 (24.4)
Atezolizumab		10 (3.6)
Nivolumab + ipilimumab		5 (1.8)
Autoimmune diseases		
Hypothyroid		4 (1.5)
Multiple arthritis		1 (0.4)

ECOG PS, Eastern Cooperative Oncology Group performance status; ICI, immune checkpoint inhibitor.

### Antibodies before introduction of ICIs

Preexisting Abs are summarized in Supplementary Table S1, available at https://doi.org/10.1016/j.esmoop.2022.100415. Of 218 patients, only 1 patient was positive for AchR Ab. The number of patients with positive ANA, Tg Ab, TPO Ab, PA-IgG Ab, and GAD Ab was 52 (24.9%), 38 (14.5%), 11 (10.1%), 6 (3.5%), and 5 (2.0%), respectively.

### irAEs and discontinuation of ICIs

Of 275 patients, 124 (45.1%) had experienced any of the irAEs. Forty-four (16.0%) presented thyroid dysfunction, 29 (10.5%) developed rash, 24 (8.7%) had interstitial pneumonitis, 17 (6.2%) had adrenal failure, 15 (5.5%) had colitis, 8 (2.9%) had hepatitis, 6 (2.2%) had renal dysfunction, 2 (0.7%) had cholangitis, 2 (0.7%) had arthritis, and 1 (0.4%) had diabetes mellitus. Seventy-three patients received the immunosuppressive or endocrine therapy in 124 patients who had irAEs (Table 2). Twenty-six (21.0%) patients had discontinued the treatment of ICI due to irAEs. More than half of the patients with interstitial pneumonitis, hepatitis, arthritis, cholangitis, or diabetes mellitus had discontinued the treatment of ICI (Table 3).

**Table 2:** The frequency of irAEs and CTCAE grade

irAE	Patients with any grade irAEs, *n* (%)	Patients who had grade ≥3 irAEs, *n* (%)
Any	124 (45.1)	—
Thyroid dysfunction	44 (16.0)	0 (0.0)
Rash	29 (10.5)	2 (0.7)
Interstitial pneumonitis	24 (8.7)	7 (2.5)
Adrenal failure	17 (6.2)	5 (1.8)
Colitis	15 (5.5)	1 (0.4)
Hepatitis	8 (2.9)	3 (1.1)
Renal dysfunction	6 (2.2)	1 (0.4)
Cholangitis	2 (0.7)	1 (0.4)
Arthritis	2 (0.7)	0 (0.0)
Diabetes mellitus	1 (0.4)	1 (0.4)

CTCAE, Common Terminology Criteria for Adverse Events; ICI, immune checkpoint inhibitor; irAE, immune-related adverse event.

**Table 3:** The frequency of ICI discontinuation

irAE (no. of patients with any grade)	Patients who discontinued ICI, *n* (%)	Patients who had grade ≥3 irAEs, *n* (%)
Any (124)	26 (21.0)	—
Thyroid dysfunction (44)	3 (6.8)	0 (0.0)
Rash (29)	5 (17.2)	0 (0.0)
Interstitial pneumonitis (24)	12 (50.0)	4 (16.7)
Adrenal failure (17)	3 (17.6)	1 (5.9)
Colitis (15)	5 (33.3)	0 (0.0)
Hepatitis (8)	5 (62.5)	3 (37.5)
Renal dysfunction (6)	2 (33.3)	1 (16.7)
Cholangitis (2)	1 (50.0)	1 (50.0)
Arthritis (2)	0 (0.0)	0 (0.0)
Diabetes mellitus (1)	1 (100)	1 (100)

ICI, immune checkpoint inhibitor; irAE, immune-related adverse event.

**Figure 1:**
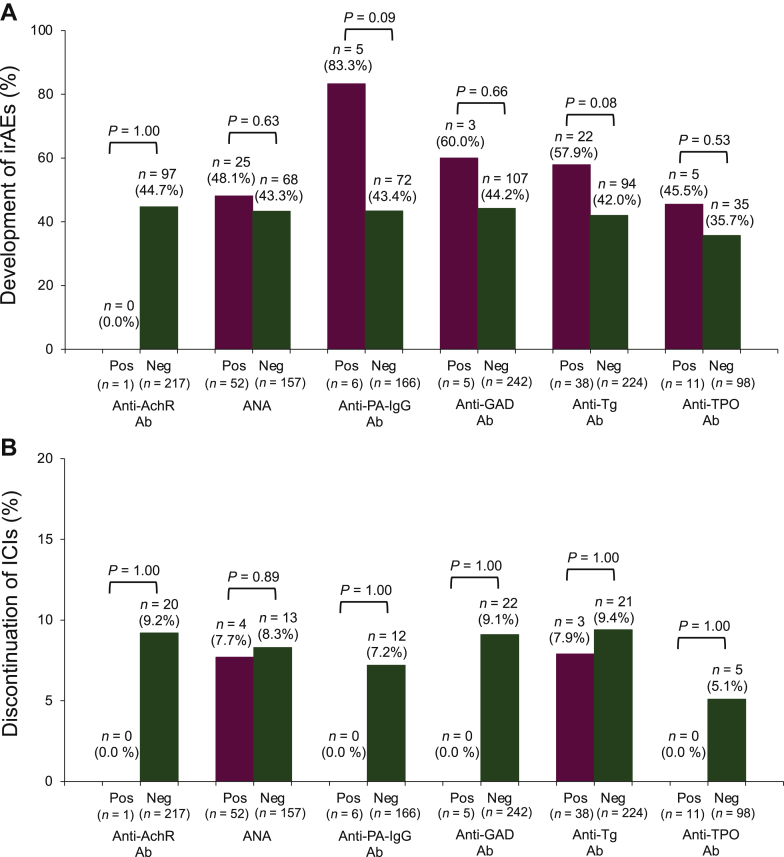
The association between irAE or discontinuation of ICIs and immune-related antibodies. The relationship between immune-related adverse events (A) or discontinuation (B) of immune checkpoint inhibitors and immune-related antibodies. AchR, acetylcholine esterase receptor; ANA, anti-nuclear antibody; GAD, glutamic acid decarboxylase; ICI, immune checkpoint inhibitor; irAE, immune-related adverse event; Neg, negative; PA, platelet-associated; Pos, positive; Tg, thyroglobulin; TPO, thyroid peroxidase.

We investigated the relationship between irAEs or discontinuation of ICI and autoimmune disease-related Abs. Patients with anti-PA-IgG Ab tended to be at a higher incidence of irAE compared with those without Ab (83.3% versus 43.4%, *P* = 0.09). There was no association between the preexisting autoimmune disease-related Abs and the development of any irAEs or the discontinuation of ICI (Figure 1).

### irAEs and immune-related antibodies

Thyroid dysfunction was observed more frequently among patients with than without preexisting Tg Ab or TPO Ab (39.5% versus 12.5%, *P* < 0.01; 45.5% versus 14.3%, *P* = 0.02). Also, interstitial pneumonitis was observed more frequently among patients with than without preexisting GAD Ab (60.0% versus 7.9%, *P* < 0.01) (Table 4). There was no association between irAEs and other preexisting autoimmune disease-related Abs. Characteristics and clinical outcomes in patients with GAD Ab are summarized in Supplementary Table S2, available at https://doi.org/10.1016/j.esmoop.2022.100415. Of five patients with GAD Ab, four patients had NSCLC, one patient had gastric cancer, no patients had a history of diabetes mellitus, and two patients discontinued the ICI therapy due to irAEs.

**Table 4:** The rate of each irAE according to status of immune-related antibodies

irAE	Patients, *n* (%)																	
	Anti-AchR Ab			ANA			Anti-PA-IgG Ab			Anti-GAD Ab			Anti-Tg Ab			Anti-TPO Ab		
	Positive (*n* = 1)	Negative (*n* = 217)	*P* value	Positive (*n* = 52)	Negative (*n* = 157)	*P* value	Positive (*n* = 6)	Negative (*n* = 166)	*P* value	Positive (*n* = 5)	Negative (*n* = 242)	*P* value	Positive (*n* = 38)	Negative (*n* = 224)	*P* value	Positive (*n* = 11)	Negative (*n* = 98)	*P* value
Thyroid dysfunction	0 (0.0)	36 (16.6)	1.00	9 (17.3)	27 (17.2)	1.00	3 (50.0)	26 (15.7)	0.06	0 (0.0)	41 (16.9)	0.59	15 (39.5)	28 (12.5)	<0.01	5 (45.5)	14 (14.3)	0.02
Rash	0 (0.0)	20 (9.2)	1.00	5 (9.6)	12 (7.6)	0.77	0 (0.0)	14 (8.4)	1.00	0 (0.0)	24 (9.9)	1.00	2 (5.3)	22 (9.8)	0.55	1 (9.0)	7 (7.1)	0.59
Interstitial pneumonitis	0 (0.0)	21 (9.7)	1.00	6 (11.5)	13 (8.3)	0.58	1 (16.7)	15 (9.0)	0.45	3 (60.0)	19 (7.9)	<0.01	4 (10.5)	19 (8.5)	0.76	0 (0.0)	8 (8.2)	1.00
Adrenal failure	0 (0.0)	12 (5.5)	1.00	2 (3.8)	10 (6.4)	0.73	1 (16.7)	10 (6.0)	0.33	1 (20.0)	16 (6.6)	0.30	1 (2.6)	16 (7.1)	0.48	1 (9.0)	6 (6.1)	0.54
Colitis	0 (0.0)	9 (4.1)	1.00	3 (5.8)	8 (5.1)	1.00	0 (0.0)	8 (4.8)	1.00	0 (0.0)	9 (3.7)	1.00	1 (2.6)	11 (4.9)	1.00	0 (0.0)	4 (4.1)	1.00
Hepatitis	0 (0.0)	7 (3.2)	1.00	2 (3.8)	6 (3.8)	1.00	0 (0.0)	7 (4.2)	1.00	0 (0.0)	7 (2.9)	1.00	3 (7.9)	5 (2.2)	0.09	0 (0.0)	2 (2.0)	1.00
Renal dysfunction	0 (0.0)	4 (1.8)	1.00	1 (1.9)	1 (0.6)	0.44	0 (0.0)	2 (1.2)	1.00	0 (0.0)	6 (2.5)	1.00	1 (2.6)	5 (2.2)	1.00	0 (0.0)	0 (0.0)	1.00
Cholangitis	0 (0.0)	2 (0.9)	1.00	0 (0.0)	1 (0.6)	1.00	0 (0.0)	1 (0.6)	1.00	0 (0.0)	2 (0.8)	1.00	0 (0.0)	2 (0.9)	1.00	0 (0.0)	0 (0.0)	1.00
Arthritis	0 (0.0)	2 (0.9)	1.00	0 (0.0)	1 (0.6)	1.00	0 (0.0)	1 (0.6)	1.00	1 (20.0)	1 (0.4)	0.04	0 (0.0)	2 (0.9)	1.00	0 (0.0)	0 (0.0)	1.00
Diabetes mellitus	0 (0.0)	1 (0.5)	1.00	0 (0.0)	1 (0.6)	1.00	0 (0.0)	1 (0.6)	1.00	0 (0.0)	1 (0.4)	1.00	0 (0.0)	1 (0.4)	1.00	0 (0.0)	1 (1.0)	1.00

AchR, acetylcholine esterase receptor; ANA, anti-nuclear antibody; GAD, glutamic acid decarboxylase; irAE, immune-related adverse event; PA, platelet associated; Tg, thyroglobulin; TPO, thyroid peroxidase.

## Discussion

To our knowledge, this is the first report to investigate the clinical utility of comprehensive measurement of autoimmune disease-related Abs, such as ANA, Tg Ab, TPO Ab, GAD Ab, AchR Ab, and PA-IgG, in cancer patients treated with ICIs. Toi et al. demonstrated that the measurement of preexisting Abs, such as RF, ANA, and anti-thyroid Abs, was a predictive tool for the development of irAEs;^2^ therefore, we expected that the comprehensive measurement of autoimmune disease-related Abs before ICI therapy was clinically helpful in predicting irAEs. Our data showed that there was no association of Abs positivity with the development of any irAEs or discontinuation of ICI therapy. However, Tg Ab and TPO Ab were associated with the occurrence of thyroid irAE and GAD Ab was associated with the occurrence of interstitial pneumonitis. Although a uniformly comprehensive measurement of the Abs before treatment with ICIs would not be clinically necessary for patients who receive ICI therapy, the measurement of Tg Ab, TPO Ab, and GAD Ab might be meaningful for predicting the development of specified irAEs.

Thyroid dysfunction is a common irAE in patients treated with ICI therapy,^8^ and is characterized by the early-onset, frequently preceded by transient hyperthyroidism followed by permanent hypothyroidism.^9^ Previous reports have shown that the development of thyroid dysfunction was significantly associated with positive Tg Ab and/or TPO Ab before ICI therapy in patients with NSCLC, melanoma, and renal cell carcinoma.^2^^,^^3^^,^^10^ Our results including various types of cancer were consistent with these previous reports. These findings suggest that thyroid Abs serve as a predictor of thyroid dysfunction induced by ICI therapy in patients with various types of cancer.

Several reports have remarkably demonstrated cases of the onset of fulminant type 1 diabetes with a presence of GAD Ab during ICI therapy.^4^^,^^11^, ^12^, ^13^ Previous reports suggest that the destruction of β-cells due to ICI therapy could cause a rapid release of the intracellular GAD antigen with the consequent development of GAD Abs.^14^^,^^15^ Alternatively, there is insufficient evidence to conclude that the presence of GAD Abs at baseline is a risk factor for type 1 diabetes in patients treated with ICI therapy. Our data showed that the occurrence of type 1 diabetes was rare; therefore, the preexisting GAD Ab before ICI therapy was not associated with the development of fulminant type 1 diabetes. Although a significant association was observed between GAD Ab and interstitial pneumonitis in our study, it is unclear on the relationship between GAD Ab positivity and occurrence of interstitial pneumonitis.

The frequency of patients with AchR Ab or PA-IgG was very low, and then, no patient had developed irAEs such as myasthenia gravis or thrombocytopenic purpura. The number of patients with ANA was 52 (24.9%), similar to that of the general population in Japan.^16^ Toi et al. reported that the development of irAE, especially skin reaction, was significantly frequent in the ANA-positive group compared with the ANA-negative group.^2^ Also, Morimoto et al. showed a tendency for increased frequency of total irAEs as well as severe irAEs, and the frequency of discontinuation of all treatment components due to severe AEs was significantly higher in the ANA-positive group than in the ANA-negative group.^17^ These results were inconsistent with our data. Hayashi et al. showed that the proportion of ANA positivity was significantly higher among females than males in the general population and approximately half of the individuals who were positive for disease-specific ANA were asymptomatic and clinically healthy.^16^ In our study, the ratio of females to males was higher than in previous studies.^2^^,^^17^ Therefore, we guess that more patients with non-disease-specific ANA might be included in this present study.

Our study had some limitations. Firstly, the frequency of Abs and the development of irAEs, such as type 1 diabetes, was very low. Therefore, the sample size was relatively small for the analyses. The database was not appropriate for the analysis by cancer type because various types of cancer were included in this study. Secondly, there was a risk for an inherent selection bias in this study due to the ambiguous definition of irAE. Finally, it was unclear whether other several Abs, such as islet-cell Abs, contributed to the development of irAEs.

### Conclusion

Our study revealed that the clinical utility of comprehensive measurement of autoimmune disease-related Abs before introduction of ICI therapy was limited for predicting irAE in cancer patients. However, the presence of Tg Ab or TPO Ab was a risk factor for the development of ICI-induced thyroid dysfunction.
