# Supplementary material for: The clinical utility of comprehensive measurement of autoimmune disease-related antibodies in patients with advanced solid tumors receiving immune checkpoint inhibitors: a retrospective study
Source: ESMO Open. 2022 Mar 2;7(2):100415. doi: 10.1016/j.esmoop.2022.100415 (PMC9058890; doi:10.1016/j.esmoop.2022.100415)
Supplement: Supplementary Tables S1 and S2 [file mmc1.docx]

Supplementary Table 1.　Positive rate of antibodies and the association with immune-related Adverse Events

|  | No. of measured Abs | No. of Abs positive |
| --- | --- | --- |
|  |  |  |
| ANA | 209 | 52 (24.9%) |
| Anti-Tg Ab | 262 | 38 (14.5%) |
| Anti-TPO Ab | 109 | 11 (10.1%) |
| Anti-PA-IgG Ab | 172 | 6 (3.5%) |
| Anti-GAD Ab | 247 | 5 (2.0%) |
| Anti-AchR Ab | 218 | 1 (0.5%) |

Abs, antibodies; ANA, antinuclear antibody; Tg, thyroglobulin; TPO, thyroid peroxidase; GAD, glutamic acid decarboxylase; AchR, acetylcholine esterase receptor; PA, platelet-associated.

Supplementary Table 2. Patients characteristics with GAD Ab

| Age | Gender | Type of cancer | GAD Ab (U/ml) | Relevant history of Autoimmune disease | ICI | irAEs (grade) | Time to onset from starting ICI (months) | Continuation of ICI |
| --- | --- | --- | --- | --- | --- | --- | --- | --- |
| 78 | male | NSCLC | 45.7 | asthma | nivolumab | - | - | - |
| 60 | male | NSCLC | 9.9 | - | nivolumab | Interstitial pneumonitis (2), Arthritis (2) | 2.3 | Continue |
| 68 | female | NSCLC | 37.4 | - | nivolumab | - | - | - |
| 49 | female | NSCLC | 6.6 | Hashimoto’s thyroiditis | nivolumab | Interstitial pneumonitis (2) | 3.1 | Discontinue |
| 76 | female | Gastric cancer | 19.3 | - | nivolumab | Interstitial pneumonitis (3), adrenal failure (2) | 7.8 | Discontinue |

NSCLC, non-small cell lung cancer; GAD Ab, glutamic acid decarboxylase antibody; ICI, immune checkpoint inhibitor; irAE, immune-related adverse event.
